# Value of Magnetic Resonance Diffusion Tensor Imaging Combined with Quantitative Electroencephalogram in Diagnosis of Neurocognitive Impairment in Patients with White Matter Demyelination

**DOI:** 10.1155/2021/2120130

**Published:** 2021-08-03

**Authors:** Jun Li, Hongtao Li, Yun Ma, Xiaowei Cai, Yinjie Zhong, Chunjie Song

**Affiliations:** ^1^Department of Neurology, Suqian First People's Hospital, Suqian 223800, Jiangsu, China; ^2^Department of Imaging, Suqian First People's Hospital, Suqian 223800, Jiangsu, China

## Abstract

This paper aimed to explore the clinical value of combined adoption of magnetic resonance diffusion tensor imaging (DTI) and quantitative electroencephalogram (QEEG) in assessing microstructure changes and mild neurocognitive dysfunction in patients with white matter demyelination. 128 cases of white matter demyelination admitted to the hospital from October 2018 to October 2019 were rolled into the research group, and 100 healthy patients physically examined during the same period were rolled into the control (ctrl) group. QEEG and magnetic resonance DTI examinations were performed for all patients. The wave power of *δ*, *θ*, *α*, and *β* and the ratio of *α*/*θ* and (*δ* + *θ*)/(*α* + *β*) were recorded. The FA values of white matter fibers in different brain areas were measured, and the Montreal Cognitive Assessment (MoCA) and Addenbrooke Cognitive Evaluation rating (ACE-R) were adopted to assess the neurocognitive function of patients. It was found that the dominant frequency of each brain area in the research group was 8-9 Hz slow *α* wave. In contrast with the ctrl, the *α* wave and *α*/*θ* values in the research group were lower, while *θ* wave and *δ* + *θ*/*α* + *β* values were higher (*P* < 0.05); the scores of ACE-R and MoCA were lower (*P* < 0.01); the fractional anisotropy (FA) values of the right frontal lobe white matter (0.335 ± 0.068), the left temporal lobe white matter (0.391 ± 0.032), and the corpus callosum knee white matter (0.658 ± 0.053) were lower (*P* < 0.05). The FA values of these three areas were positively correlated with attention and calculation, memory, and memory of MoCA scale, respectively (*P* < 0.05). The FA value of the right frontal white matter was positively correlated with the attention and calculation score of the ACE-R scale (*P* < 0.05). In conclusion, magnetic resonance DTI combined with QEEG could reflect the microstructural changes of white matter, which may be associated with mild neurocognitive impairment. The primary objective of the study was to explore the clinical value of combined adoption of magnetic resonance DTI and QEEG in assessing microstructure changes and mild neurocognitive dysfunction in patients with white matter demyelination, expected to provide a theoretical basis for the treatment of white matter demyelination.

## 1. Introduction

White matter lesions mostly occur in the elderly over 50 years old, and their imaging examinations usually show white matter hyperintensity [1]. The causes of white matter mainly include genetics, hydrocephalus, poisoning, infection, and other factors. Neuropathologically, demyelination, cell edema and apoptosis, axon damage, and glial cell proliferation all can cause white matter lesions [2]. The white matter demyelination is a secondary manifestation of neurological diseases such as senile degenerative changes and ischemic infarction. After the occurrence of these neurological diseases, the normally developed myelin sheath appears to have myelin sheath loss and necrosis [3]. Usually, there are sclerosing, vascular, dystrophic demyelination, and so on. Patients with white matter demyelination will experience a series of mental symptoms, visual disturbances, and language disturbances after the onset. Studies have shown that the white matter can cause cognitive dysfunction, and if the disease is serious, it may develop into dementia [4, 5]. Because the white matter demyelination has a strong volatility, usually there is a recurrence. It may also cause human dysfunction, which in turn makes the condition worse. Therefore, early diagnosis and early treatment is required for white matter demyelination, and timely diagnosis can improve the prognosis, thereby reducing the pain of the disease and prolonging the survival time.

Electrophysiological examination is an effective way to study brain function. QEEG is a new research method under the rapid development of computer technology, and the main core technology is power spectrum analysis [6]. First, conventional brain wave data are collected. Then, Fourier transform is performed on the EEG signal, to convert the previous time domain signal into the frequency domain. Finally, the spectrogram is obtained. Studies have shown that QEEG has achieved good results in the evaluation of neurocognitive function in patients with Parkinson's and Alzheimer's [7]. Montreal cognitive assessment (MoCA) is a cognitive assessment tool widely used in clinical practice worldwide. Addenbrooke cognitive evaluation rating (ACE-R) is a scale for mild cognitive impairment, and it is widely used in early screening of cognitive dysfunction [8]. DTI is a noninvasive MRI technology that can clearly observe the movement of water molecules in the tissue and diffusion in different directions. Besides, it can also provide information that other conventional technologies cannot provide, such as nerve fiber orientation and nerve damage and organizational microstructure [9]. DTI is effective in the evaluation of the pathophysiological changes of tissue structure. Therefore, it is widely used in the research of central nervous system diseases [10]. However, there are not many studies on white matter demyelination. In the study, the clinical value of magnetic resonance DTI combined with QEEG was explored in evaluating microstructure changes and mild neurocognitive dysfunction in patients with white matter demyelination.

## 2. Materials and Methods

### 2.1. Research Subjects

128 cases of white matter demyelination, admitted to our hospital from October 2018 to October 2019, were rolled into the research group, and 100 healthy patients during the same period were rolled into ctrl. In the research group, there were 78 males and 50 females; in the ctrl, there were 62 males and 38 females, and there were no obvious differences between the two groups (*P* > 0.05). This experiment has got permission from the ethics committee of the hospital, and the patients included in the study were aware of and agreed to it.

Inclusion criteria of the research group: (i) patients with DTI examination; (ii) patients with QEEG examination; (iii) patients diagnosed with white matter demyelination by imaging; and (iv) patients with cognitive dysfunction. Exclusion criteria: (i) patients with other neurological diseases that would cause neurological dysfunction; (ii) patients with heart, liver, and kidney dysfunction; (iii) patients with thyroid dysfunction; (iv) patients with a mental illness history; (v) patients taking cognitive drugs within the past month; and (vi) patients who cannot have DTI inspection. Inclusion criteria for the ctrl: (i) patients with no cognitive decline; (ii) patients whose Hamilton depression scores were less than 8 points; (iii) patients with a history of mental illnesses or patients unwilling to cooperate to conduct the research; (iv) patients whose Clinical Dementia Scale (CDR) scores were 0; and (v) patients with Daily Activity Score (ADL) less than 26 points.

### 2.2. QEEG Detection

The tracing was started in a quiet and semidark room environment. The subject sat in a chair, remained awake, closed his eyes, and relaxed during the entire examination. According to the international 10–20 system, a total of 16 Ag-AgCl disc electrodes were placed on the subject's scalp (5 sites of F3, C3, P3, O1, and T3 were selected for subsequent analysis). Reference electrodes were put at the left and right earlobes. Then, monopolar lead tracing began. Tracing was done more than 15 minutes, the time constant was 0.3, the gain was 100 *µ*V, and the high frequency filter was 25 Hz. The EEGs without eye movement for 30 seconds were selected, and the power values were obtained through the analog-digital fast Fourier conversion. Then, the power spectrum of *δ*, *θ*, and *α* wave and the ratio values of *α*/*θ*, (*δ* + *θ*)/(*α* + *β*) were calculated. The frequencies of *δ*, *θ*, *α*, and *β* waves were 1.0–3.9, 4.0–7.9, 8.0–13.9, and 14.0–30.0 Hz, respectively.

### 2.3. Magnetic Resonance DTI

All subjects selected underwent routine MRI and DTI imaging examinations. First, conventional MRI scan was performed. Sagittal FRFSE T2W1 was adopted, and TR and TE were 3600 ms and 100.4 ms, respectively. For axial FSE TIFLAIR, TR, TE, and TI were 1700 ms, 28.2 ms, and 750 ms, respectively. For axial FSE T2W1, TR and TE were 5500 ms and 120.2 ms, respectively. For T2FLAIR sequence, TR, TE, and TI were 8000 ms, 135 ms, and 2000 ms, respectively. The layer thickness was 5 mm, the layer distance was 1 mm, the FOV was 24  cm × 24  cm, and the matrix was 28 × 28. The main purpose of scanning was to rule out hydrocephalus, intracerebral swelling, multiple cerebral infarction, and other diseases. DTI imaging scan parameter setting: single-shot SE/EPI sequence was adopted. The slice thickness was 4 mm, slice distance was 0 mm, and TR and TE were 5500 ms and 92.4 ms, respectively. Besides, FOV and matrix were the same as conventional MRI, and the B value was 0 and 1000 s/mm^2^. There were 25 nonlinear directions in total, and the scanning plane was parallel to the front-back joint line, namely, the AC-PC line.

**Figure 1:**
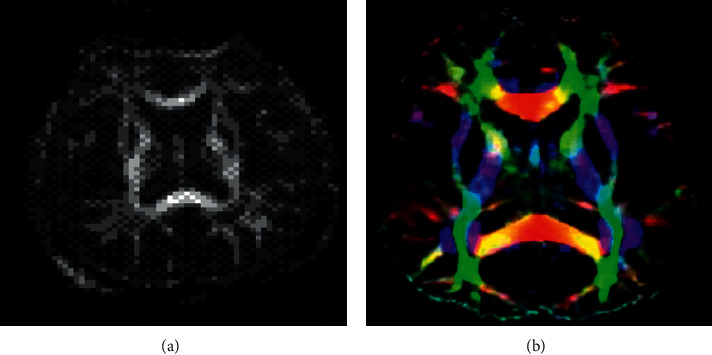
FA measurement and DTI fiber tracking. (a) FA measurement map. (b) Color map of DTI fiber tracking.

After the original image was scanned, it was uploaded to the workstation and processed by FA smooth image reconstruction and Fiber Trak software. As a result, the FA color image was obtained (Figure 1). About 20 mm^2^ ROI was selected to measure the white matter fiber FA value on the FA map of T2W1 EPI.

### 2.4. Neurocognitive Function Assessment

ACE-R [11] and MoCA [12] were used to evaluate the neurocognitive function on the first day of admission. ACE-R is a comprehensive cognitive assessment scale designed and revised by the University of Cambridge based on a large amount of experience. It has extremely high sensitivity and specificity at the critical point of diagnosis of dementia, 83/100 and 88/100. It evaluates factoring into five aspects: memory (26), language fluency (14 points), attention or orientation (18 points), visual space (16 points), and language (26 points). The total score is 100 points, and a higher value indicates better cognitive effects.

### 2.5. Statistical Analysis

Data were processed by SPSS20.0, mean ± standard deviation ($\bar{x}\pm s$) was how measurement data were expressed, count data was expressed as percentage, using *t*-test or *χ*^2^ test. The FA value, MoCA, and ACE-R scores were used for canonical correlation analysis. If *P* < 0.05, it was considered to have a statistically significant difference.

## 3. Results

### 3.1. General Data of Research Subjects

128 cases of white matter demyelination, admitted to our hospital from October 2018 to October 2019, were rolled into the research group, and 100 healthy patients during the same period were rolled into the ctrl. In the research group, there were 78 males and 50 females; in the ctrl, there were 62 males and 38 females; and there were no obvious differences between the two groups (*P* > 0.05). This experiment has got permission from the ethics committee of our hospital, and the patients included in the study were aware of and agreed to it (Table 1).

### 3.2. QEEG Power Spectrum Analysis

**Figure 2:**
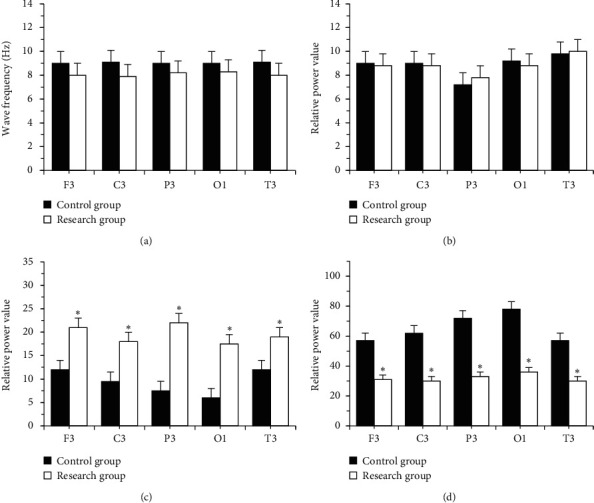
Dominant frequency distribution and power comparison. (a) Main frequency distribution. (b) Delta wave. (c) *θ* wave. (d) Alpha wave. ^*∗*^ indicated that, in contrast with the research group, *P* < 0.05 (with the same meaning in Figures 3–6).

**Figure 3:**
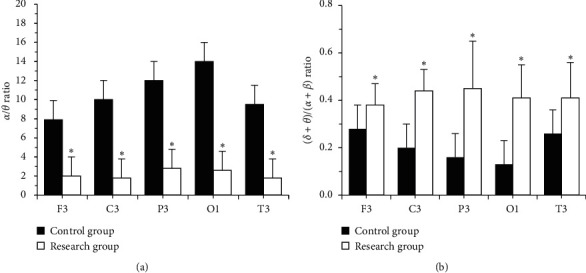
Values of *α*/*θ* and (*δ* + *θ*)/(*α* + *β*). (a) The value of *α*/*θ*. (b) The value of (*δ* + *θ*)/(*α* + *β*)).

The dominant frequency of each brain area in the research group was 8-9 Hz slow alpha wave (*P* > 0.05). The relative power of delta waves between the two groups was not much different (*P* > 0.05). In contrast with the ctrl, the values of *α* wave and *α*/*θ* in the research group were obviously lower (*P* < 0.05), and the values of theta wave and *δ* + *θ*/*α* + *β* were obviously higher (*P* < 0.05) (Figures 2 and 3).

### 3.3. Comparison of White Matter DTI

**Figure 4:**
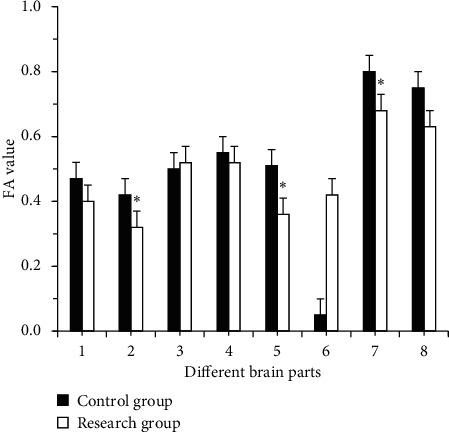
Comparison of FA values (1: left frontal white matter; 2: right frontal white matter; 3: left parietal white matter; 4: right parietal white matter; 5: left temporal lobe white matter; 6: right temporal lobe white matter; 7: the knee of the corpus callosum; 8: the pressure of the corpus callosum).

The FA values of the right frontal lobe white matter (0.335 ± 0.068), the left temporal lobe white matter (0.391 ± 0.032), and the corpus callosum knee (0.658 ± 0.053) of the research group were lower than the ctrl (*P* < 0.05), and there were no obvious differences in the other parts (Figure 4).

### 3.4. MoCA and SPMSQ Scores

The MoCA and ACE-R scores in the research group were 20.23 ± 2.65 (points) and 28.12 ± 2.54 (points); and those in the ctrl were 25.34 ± 3.27 (points) and 23.98 ± 4.08 (points), respectively. Obviously, the ACE-R score in the research group was lower versus the control group (*P* < 0.01) (Figure 7).

**Figure 5:**
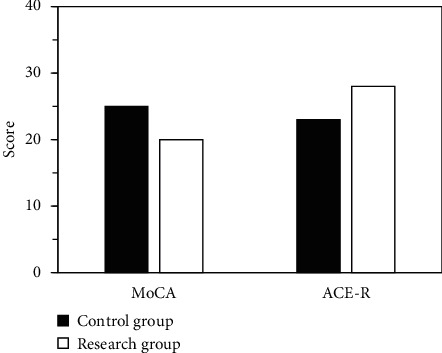
Comparison of MoCA and ACE-R scores (^*∗∗*^ indicated that, in contrast with the ctrl, *P* < 0.01 (with the same meanings in Figures 5 and 6)).

The comparison of each dimension of the MoCA scale between the two groups is shown in Figure 5. The difference in naming, visual space, and the execution of the two were not obvious (*P* > 0.05). In contrast with the ctrl, the language and abstract thinking of the research group were obviously lower (*P* < 0.05), and the memory, orientation, attention, and calculation skills were obviously lower (*P* < 0.01).

**Figure 6:**
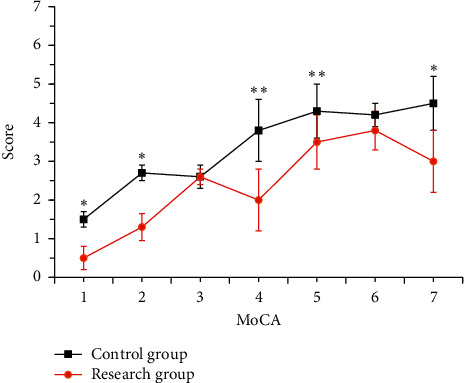
Comparison of the MoCA scores (1: abstract thinking; 2: language; 3: naming; 4: orientation; 5: directional force; 6: visual space and execution; 7: attention and calculation).

**Figure 7:**
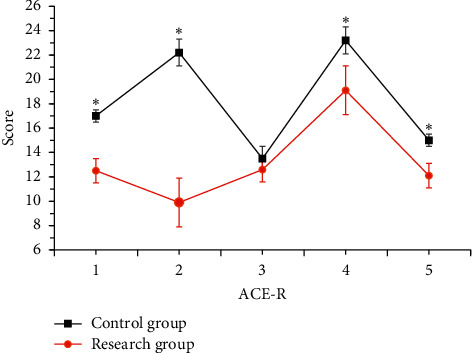
Comparison of ACE-R scores (*Note.*^*∗*^ indicated an obvious difference, *P* < 0.05, 1: attention and orientation; 2: memory; 3: language fluency; 4: visual space; and 5: language).

The comparison of the scores of each dimension of the ACE-R scale between the two groups is shown in Figure 6. There were obvious differences in memory, attention and orientation, and visual space between the groups (*P* < 0.05). It was evident from the figure that memory had the most obvious decrease.

### 3.5. Correlation Analysis

The canonical correlation analysis was adopted to analyze the relationship between FA value and the scores of each dimension of the MoCA scale in the research group (Table 2). It was evident that the FA value of the right frontal lobe white matter was positively correlated with the attention and calculation score of the MoCA scale (*R* = 0.315, *P*=0.036), the FA value of the left temporal lobe white matter was positively correlated with the memory score of the MoCA scale (*R* = 0.432, *P*=0.021), and the FA value of the corpus callosum was positively correlated with the memory score of the MoCA scale (*R* = 0.423, *P*=0.019).

The canonical correlation analysis was adopted (Table 3). It was evident that the FA value of the right frontal lobe white matter was positively correlated with the attention and calculation score of the ACE-R scale (*R* = 0.332, *P*=0.017).

## 4. Discussion

As the aging of the population accelerates, the incidence and diagnosis rate of white matter lesions are increasing year by year. White matter lesions are malignant and clinically manifest as cognitive decline or development of dementia. The primary objective of the study was to explore the clinical value of magnetic resonance DTI combined with QEEG in assessing microstructure changes and mild neurocognitive dysfunction in patients with white matter demyelination. QEEG was used to detect white matter demyelinating lesions. It was found that the dominant frequency of each brain area in the research group was 8-9 Hz slow alpha wave, with no obvious difference in contrast with the ctrl (*P* > 0.05). The relative powers of *δ* waves of the two groups were not much different (*P* < 0.05). In contrast with the ctrl, *α* wave and *α*/*θ* values of the research group were obviously lower (*P* < 0.05), and *δ* + *θ*/*α* + *β* was obviously higher (*P* < 0.05). It can be inferred that patients with white matter demyelinating have insignificant changes in dominant frequency distribution and delta wave power, a decrease in *α* wave power and *a*/*θ* value, and an increase in *θ* wave and *δ* + *θ*/*α* + *β* value. Therefore, the power of *α* wave, the value of *α*/*θ*, the value of *θ* wave, and *δ* + *θ*/*α* + *β* were QEEG indicators with auxiliary diagnostic value [13]. The scores of the SPMSQ scale showed that the difference in learning, language, and execution of the two groups was not obvious (*P* > 0.05). In contrast with the ctrl, the graphic copying, attention, and calculation ability of the research group was lower (*P* < 0.05), and the memory was obviously lower (*P* < 0.01). It may be because the white matter demyelinating leads to short-term memory impairment, which was in line with the results of Qi et al. [14]. The MoCA scale contains more complex test items, which can effectively evaluate the severity of patients' lesions. The scores of the MoCA scale showed that the difference in naming, visual space, and the execution of the two groups were not obvious (*P* > 0.05). In contrast with the ctrl, the language and abstract thinking of the research group were lower (*P* < 0.05), and memory, orientation, attention, and calculation were obviously lower (*P* < 0.01). Ritchie et al. found that the MoCA scale had a sensitivity of 80.48% in diagnosing cognitive dysfunction and a specificity of 81.19% [15]. It was in line with the results of the study. Based on MoCA scores, Gaete et al. [16] found that, age and educational level were significantly associated with the overall cognition level. It was found in the study that the FA value of the right frontal lobe white matter was positively correlated with the attention and calculation score (*R* = 0.315, *P*=0.036), the FA value of left temporal lobe white matter was positively correlated with the memory score (*R* = 0.432, *P*=0.021), and the FA value of the knees of the corpus callosum was positively correlated with the memory score (*R* = 0.423, *P*=0.019). It was consistent with the results of Bledsoe et al. [17] and Elahi et al. [18]. Nevertheless, the selection of subjects in this study is limited by region and time, and the sample size is small. In the follow-up, an expanded sample size is necessary to analyze the microstructure changes and neurological recognition of patients with white matter demyelination.

## 5. Conclusion

White matter demyelination can cause cognitive dysfunction. The combined application of magnetic resonance DTI and QEEG can provide an important theoretical basis for the clinical diagnosis and treatment of white matter demyelination. However, the number of samples in this article is limited, which needs to be increased in the future. Furthermore, only the FA value of the DTI technology is discussed. Later, the analysis of other parameters such as the average or radial diffusion coefficient can be added for in-depth study. In short, the results provide a theoretical basis for the clinical diagnosis and follow-up treatment of patients with demyelinating white matter.

## Figures and Tables

**Table 1:** General data comparison.

	Ctrl (*n* = 100)	Research group (*n* = 128)	*P*
Gender (male female)	62/38	78/50	0.398
Age (year)	67.23 ± 6.78	67.52 ± 7.81	0.423
Education years (year)	12.76 ± 5.28	13.09 ± 5.17	0.725

**Table 2:** Correlation analysis between FA value with MoCA.

	FA value of the right frontal white matter		FA value of the left temporal lobe white matter		FA value of corpus callosum of knee	
	*R*	*P*	*R*	*P*	*R*	*P*
Language	−0.423	0.130	−0.655	0.724	0.651	0.083
Memory	−0.752	0.106	0.432	0.021^*∗*^	0.423	0.019^*∗*^
Orientation	0.326	0.215	0.532	0.221	−0.319	0.472
Abstract thinking	−0.327	0.634	−0.487	0.082	−0.573	0.326
Attention and calculation	0.315	0.036^*∗*^	−0.516	0.068	0.416	0.054

*Note.*^*∗*^ meant *P* < 0.05.

**Table 3:** Correlation analysis between FA values with ACE-R.

	FA value of the right frontal white matter		FA value of the left temporal lobe white matter		FA value of corpus callosum of knee	
	*R*	*P*	*R*	*P*	*R*	*P*
Graphic copy	−0.791	0.078	−0.743	0.118	−0.312	0.427
Memory	−0.426	0.054	−0.677	0.765	0.643	0.075
Orientation	0.328	0.225	0.553	0.208	−0.332	0.085
Attention and calculation	0.332	0.017^*∗*^	−0.406	0.327	0.137	0.126

*Note.*^*∗*^ meant *P* < 0.05.

## Data Availability

No data were used to support this study.
